# Hybrid liposome–erythrocyte drug delivery system for tumor therapy with enhanced targeting and blood circulation

**DOI:** 10.1093/rb/rbad045

**Published:** 2023-04-26

**Authors:** Kehui Zhu, Yingcan Xu, Rui Zhong, Wanjing Li, Hong Wang, Yee Shan Wong, Subramanian Venkatraman, Jiaxin Liu, Ye Cao

**Affiliations:** Institute of Blood Transfusion, Chinese Academy of Medical Sciences & Peking Union Medical College, Chengdu, China; Institute of Blood Transfusion, Chinese Academy of Medical Sciences & Peking Union Medical College, Chengdu, China; Institute of Blood Transfusion, Chinese Academy of Medical Sciences & Peking Union Medical College, Chengdu, China; Institute of Blood Transfusion, Chinese Academy of Medical Sciences & Peking Union Medical College, Chengdu, China; Institute of Blood Transfusion, Chinese Academy of Medical Sciences & Peking Union Medical College, Chengdu, China; Biomedical Engineering, School of Engineering, Temasek Polytechnic, Singapore, Singapore; School of Materials Science and Engineering, National University of Singapore, Singapore, Singapore; Institute of Blood Transfusion, Chinese Academy of Medical Sciences & Peking Union Medical College, Chengdu, China; Institute of Blood Transfusion, Chinese Academy of Medical Sciences & Peking Union Medical College, Chengdu, China

**Keywords:** liposomes, erythrocyte, RBC-hitchhiking, blood circulation, target delivery

## Abstract

Liposome, a widely used drug delivery system (DDS), still shows several disadvantages such as dominant clearance by liver and poor target organ deposition. To overcome the drawbacks of liposomes, we developed a novel red blood cell (RBC)–liposome combined DDS to modulate the tumor accumulation and extend the blood circulation life of the existing liposomal DDS. Here, RBCs, an ideal natural carrier DDS, were utilized to carry liposomes and avoid them undergo the fast clearance in the blood. In this study, liposomes could either absorbed onto RBCs’ surface or fuse with RBCs’ membrane by merely altering the interaction time at 37°C, while the interaction between liposome and RBCs would not affect RBCs’ characteristics. In the *in vivo* antitumor therapeutic efficacy study, 1,2-dipalmitoyl-sn-glycero-3-phosphocholine (DPPC) liposomes attached onto RBCs’ surfaces exhibited lung targeting effect (via RBC-hitchhiking approach) and reduced clearance in the liver, while DPPC liposomes fused with RBCs had prolong blood circulation up to 48 h and no enrichment in any organ. Furthermore, 20 mol% of DPPC liposomes were replaced with pH-sensitive phospholipid 1,2-dioleoyl-Sn-glycero-3-phosphoethanolamine (DOPE) as it could respond to the low pH tumor microenvironment and then accumulate in the tumor. The DOPE attached/fusion RBCs showed partial enrichment in lung and about 5–8% tumor accumulation, which were significantly higher than (about 0.7%) the conventional liposomal DDS. Thus, RBC–liposome composite DDS is able to improve the liposomal tumor accumulation and blood circulation and shows the clinical application promises of using autologous RBCs for antitumor therapy.

## Graphical abstract

**Figure:**
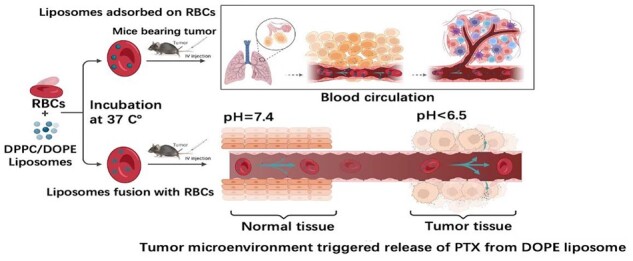

## Introduction

Liposomes are arguably the best characterized and the widely clinically used nano drug delivery systems (DDS) [1]. Despite apparent advantages, the clinical utilization of the liposomes is hindered due to some limitations [2]. One of the major limitations is the rapid clearance by the phagocytes in reticuloendothelial system (RES), which caused their enrichment in liver and spleen, resulting in low efficacy *in vivo* and the need for more frequent administrations [3–5]. To address this issue, liposomes were decorated with polyethylene glycol (PEG) molecules to provide better steric stability, longer blood circulation half-life and lower immunogenicity and toxicity to the liver [6]. Despite that, the pegylated liposomal system still shows some drawbacks, including lowered cellular uptake and accelerated blood clearance upon repeated administration due to immune memory [7–9]. Another limitation of liposome is their poor targeting of the desired cells or tissue. In a review by Stefan Wihelm *et al.* [10], only 0.7% of the drug-encapsulated liposome could be accumulated in tumor. Thus, reducing the off-target drug toxicity of liposomes is crucial for the therapeutic efficiency improvement. Several studies [11, 12] have explored the attachment of affinity moieties, such as antibodies and their fragments, to the surface of liposomes for the enhanced targeting ability and improved intracellular delivery efficiency. But these modifications to liposomes may cause other problems, such as unintended effects on host defense (e.g. caused by IgG multiplexed on liposomes), drug leakage during modification or the aggregation of liposomes after modification [13, 14]. Overcoming these challenges with a technically straightforward and translatable approach for desired liposomes remains the raison detre of nanomedicine research.

To overcome the above challenges, various research teams have explored the idea of combing nanomedicine with cell therapy, in particular, red blood cells (RBCs)-based DDS [15]. RBC represents an ideal carrier for vascular drug delivery as RBCs are easily available, have long circulation half-life, clinical safety for transfusion and prior clinical success in clinical trials [16, 17]. Different from the traditional harsh method (hypotonic way [18]) of loading drugs into the internal cavity of RBCs, RBC-hitchhiking (RH) technology is a relatively new approach for DDS with better RBCs compatibility [19]. RH technology, as applied to lung targeting, essentially contains three steps [20–22]: (i) the nanoparticles (NPs) attached to RBCs surface during the incubation *ex vivo* process, (ii) followed by NP detachment from RBCs when RBCs squeeze through the lung capillaries and (iii) then NPs transferred to the pulmonary capillary endothelial cells. Although the amount of RH-liposomes accumulation after intravenous (IV) administration could be enhanced to 30% of the initial dose in the lungs at 1 h, most of the liposomes were still cleared by the RES as the liposomes would be flushed from the vascular lumen by the blood stream. In the study, the NP amount in the lung was maximal at 1 h post injection and sustained at a high level for about 10 h [23].

To enhance the lung uptake of NPs, an endothelial epitope, platelet endothelial cell adsorption molecule (PECAM) was coated to NPs attached to the surface of RBCs. PECAM adsorbed RH-NPs prolonged the residence of NPs in the lungs, because the PECAM improved the binding and internalization of NPs in the lung endothelium. However, the coating of antibody to NPs-RBCs may have unintended effects on host defense, as well as causing changes to the RBC surface and affecting the RBCs half-life in blood [24, 25]. Thus, NPs-RBCs DDS could be optimized to further reduce the off-target toxicity of various drugs, enabling prolonged blood circulation and beneficial drug biodistribution.

Another challenge for RBC DDS is a safety issue: loading the NPs to RBCs may alter the RBCs themselves. For instance, the process of attaching NPs to RBCs may lead to the loss of hemoglobin, decrease in the deformability, resilience and structural integrity of the membrane; additionally, the loss of surface glycoproteins that protect RBCs from complement and phagocytosis (e.g. CD47), as well as the surface exposure of entities normally localized in the inner leaflet of the membrane (e.g. phosphatidyl serine [PS]) may cause the RBCs to undergo fast clearance by the RES [5, 26]. Uncontrolled combination of NPs to RBCs results in the uptake by phagocytes in the RES and accelerated clearance, which impedes the efficacy of RBC DDS. Thus, it is important to investigate the change in RBCs after attachment of NPs [27].

Since the liposomes have a similar structure as the cell membrane, the liposomes may attach to the RBC surface or fuse with the RBC membrane [28, 29]. Inspired by this natural property, we have developed two kinds of liposome–RBCs combination DDS, while the combination modes (attachment or fusion) between liposomes and RBCs were modulated by the incubation condition *ex vivo*. In this study, we prepared two types of liposomes: 1,2-dipalmitoyl-sn-glycero-3-phosphocholine (DPPC) liposomes and pH-responsive 1,2-dioleoyl-Sn-glycero-3-phosphoethanolamine (DOPE) liposomes. DOPE is a cationic lipid, the pH of tumor microenvironment is weakly acidic, which can trigger the protonation of DOPE, that then causes the destabilization of DOPE liposomes, which destroys the stability of the lipid bilayer and releases drugs, thus obtaining the ability of passive tumor targeting [30].

Paclitaxel (PTX) was selected as the model drug to verify the effectiveness of the erythrocytic–liposome combined DDS *in vivo*. The antitumor efficacy, their blood circulation half-life and biodistribution of erythrocytic–liposome combined DDS were evaluated in the C57 mice bearing transplanted subcutaneous Lewis lung cancer (LLC) cell sarcoma. In addition to studying the efficacy of the combination liposomes/RBCs, the adverse effects of RBCs such as induction of hemolysis, PS exposure, CD47 loss and RBC morphology and deformability were also studied. Interestingly, our results showed that DPPC liposomes fused with RBCs did not accumulate in any organs for first the 48 h and had the longest half-time in blood among all the groups. And DOPE liposomes attached or fused with RBCs could achieve tumor-targeted delivery. Both DPPC and DOPE liposomes attached to RBCs (RH technique) can partially accumulate in the lung, but they accumulated less in the liver and spleen compared with conventional DPPC/DOPE liposome groups. We demonstrated that both DPPC and DOPE liposomes in fusion with RBCs could significantly slow down the growth of lung cancer, but there was no significant difference between them. Our study proved that the antitumor therapy efficacy is a synergistic effect of both long blood circulation and tumor accumulation ability. Thus, DPPC-RBC DDS may be a promising therapeutic way for hematologic tumors, while DOPE-RBC DDS may be a good candidate for the therapy of solid tumor.

## Materials and methods

### Preparation of the PTX-liposomes

Liposomes were prepared by the conventional thin-film method. The DPPC-liposome (Lipo-DPPC) lipid bilayer was composed of DPPC and cholesterol with a molar ratio of 90:10; the DOPE-liposome (Lipo-DOPE) lipid bilayer was composed of DPPC, DOPE and cholesterol with a molar ratio of 70:20:10, resulting in the total molar amount of 18 mM. PTX (1.8 mg) embedded lipid thin film was prepared by the rotary evaporator at 45°C for 1 h, hydrated with PBS for 20 min at 45°C and then extruded through 0.2 μm and 0.1 μm polycarbonate membranes, respectively. DPPC and DOPE were obtained from AVT Pharmaceutical Tech Co. Ltd. (Shanghai) and cholesterol was obtained from Aladdin. The size of these liposomes was verified by Delsa Nano C Particle Analyzer (Beckman Coulter, Inc) based on the dynamic light scattering (DLS) method.

### Drug encapsulation efficiency

The encapsulation efficiency of PTX was determined by reverse-phase high-performance liquid chromatography system (Agilent Technologies, USA) using SB-C18 column (Agilent, 20RBSX, 5 μm, 4.6 × 150 mm). The mobile phase is composed of 39% of double distilled water, 37% of acetonitrile and 24% of methanol with a flow rate of 0.8 ml/min. To measure the PTX in liposomes and RBC-lipo, 100 μl of each sample was mixed with 400 μl acetonitrile, vortexed for 2 min and then centrifuged with 10 000×g for 2 min. The supernatant was filtered through a 0.22-μm filter and the drug-encapsulated amount was measured. Equation (1) was used to calculate the encapsulation efficiency (EE %):


(1) $$\mathrm{EE}\;\left(\%\right)=\frac{\mathrm{Amount}\;\mathrm{of}\;\mathrm{PTX}\;\mathrm{encapsulated}\;}{\mathrm{Total}\;\mathrm{amount}\;\mathrm{of}\;\mathrm{PTX}}\times 100\%.$$

### Incubation of blood in the presence of liposomes

Human blood was obtained from Chengdu Blood Center. Venous blood was collected from healthy adults, with informed consent. In total, 400 ml of whole blood was anticoagulated with 56 ml citrate–phosphate–dextrose. The leukocytes were removed by the leukocytes filter (Chengdu Shuanglu Medical Company). Leukocyte-depleted whole blood was centrifuged at 3500× g for 10 min, and then RBCs were collected for the following experiments: RBCs (2–3 × 10^8^) washed by PBS were incubated in the presence of liposomes (1–3 μmol) for 1 or 4 h at 37°C, the suspension was gently rotated (200 rpm) during incubation. Unbound liposomes were separated from RBCs by centrifugation (3500× g, 10 min).

### Scanning electron microscopic

Scanning electron microscopic (SEM) studies of RBCs were performed using a Quanta 250 electron microscope (FEI Company, Hillsboro, OR, USA). Samples were fixed in 2.5% glutaraldehyde for 2 h, mounted on mica slides, washed twice in PBS (pH 7.4), and then covered with a gold–palladium layer, the samples underwent SEM analysis.

### Confocal laser scanning microscopy

The liposomes combined with RBCs were observed by confocal laser scanning microscopy (CLSM) (ZEISS LSM 900 with Airyscan 2). FITC-tagged liposomes were prepared by the same method mentioned above with the addition of 0.2% mol 1,2-dioleoyl-sn-glycerol-3-phosphoethanolamine-*N*-(carboxyfluorescein) (ammonium salt) (Avanti, USA); DiI-labeled RBCs were obtained by incubating RBCs with DiI dye at 37°C for 15 min and washing by PBS. DiI-labeled RBCs and FITC-tagged liposomes were incubated for 1/4 h. The samples were mounted on the glass slide, washed gently with PBS, and then observed by CLSM.

### Fluorescence resonance energy transfer

The combination process between liposomes and RBCs was monitored by the dilution assay of two fluorescent lipid probes, based on resonance energy transfer. Briefly, donor fluorescent liposomes were prepared containing an equal molar ratio (0.5%) of the fluorescent lipids NBD-PE and Rh-PE (Avanti, USA), respectively. Upon excitation of NBD-PE, energy is transferred to Rh-PE in a fluorescence resonance energy transfer (FRET) process, which is strongly dependent on the distance between the two fluorophores. When adsorption or fusion occurs, the resulting dilution reduces the surface density of the fluorescent probe, resulting in a decrease in efficiency for resonance energy transfer, followed by an increase in NBD (energy donor) fluorescent intensity. The total volume of the reaction mixtures was 120 μl. Liposomes and RBCs were mixed at a liposome/RBC ratio of 2 μmol/3 × 10^8^ in PBS, liposomes and RBCs were mixed in 96-well plates maintained at 37°C in the microplate reader. The combination was monitored by continuously measuring the energy transfer from NBD-PE to Rh-PE at fluorescence excitation and emission wavelengths of 464 and 522 nm, respectively, for 6 h. The percentage of the combination was calculated according to Equation (2): where *F_t_* is the fluorescence intensity at a certain time point (*t*), *F*_0_ is the fluorescence intensity before fusion takes place (“zero” time) and *F*_100_ indicates the fluorescence intensity at the end point.


(2) $$\text{Combination efficiency}（\%）=\left(F_{t}-F_{0}\right)/\left(F_{100}-F_{0}\right)\times 100\%,$$

### Cell culture

LLCs (FuHeng Biology, Shanghai) were incubated in high glucose Dulbecco’s Modified Eagle Medium (DMEM, HyClone), containing 10% fetal bovine serum (Transgen Biotech) and 1% penicillin–streptomycin. Cell lines were cultured in a humid atmosphere with 5% CO_2_ at 37°C and subcultured every 2 days.

### Hemolysis

When the incubation of RBCs with liposomes (Lipo-DPPC/DOPE) was completed, it was centrifuged at 3000× *g*, and the supernatant was taken to detect the concentration of Hb, proceed as described for the plasma-free hemoglobin assay kit (REAL-TECH).

### RBCs osmotic fragility

Osmotic fragility was assessed using a series of diluted sodium chloride solutions with concentrations ranging from 0.1% to 0.9%. Briefly, 50 μl of each untreated RBCs and liposomes combined RBCs was added to 1 ml of each diluted sodium chloride solution and incubated at room temperature for 30 min, followed by centrifugation at 3500 × *g* for 10 min. The supernatants were collected and hemolysis was measured by a microplate reader (Bio-Tek Cytation 3) at a wavelength of 540 nm. To calculate the concentration that produced 50% hemolysis, the percent of hemolysis in each sodium chloride solution was plotted against the sodium chloride solution concentration.

### RBC deformability

RBC deformability was measured using a laser-diffraction Ektacytometer (LBY-BX, Beijing Precil Instrument Co., Ltd, China). RBCs were diluted 1:100 in a polyvinylpyrrolidone solution (0.15 g/ml) and subjected to increasing shear stress at 37°C. The shear rate was increased by a faster rotation of the outer cup to cause RBCs deformation, and erythrocytes deformability (Elongation index, EI) was detected at four different shearing forces (50, 100, 200, 1000, and 2000 Pa) according to the laser diffraction principle.

### Assessment of PS exposure and CD47 expression on RBCs

Phosphatidylserine (PS) exposure on RBCs surface and RBC-lipo was measured according to a flow cytometry procedure based on the binding of Annexin V to PS. Briefly, 2 × 10^7^ RBCs or RBC-lipo were suspended in PBS and incubated with 5 μl of Annexin V-FITC (BD Pharmingen™) or CD47 (BD Pharmingen™) for 15 min in the dark, respectively. Subsequently, the sample was resuspended in 400 ml of PBS (pH = 7.4) and detected by flow cytometry (Cytoflex, Beckman Coulter, Inc.).

### *In vivo* animal study

The 5 × 10^6^ LLC cells were washed and resuspended in PBS, which was then inoculated subcutaneously into the right flank of the mice. Tumor size was assessed using a digital caliper every day and approximate tumor volume (mm^3^) was calculated as length × width^2^/2. Treatment started when tumors were 90–110 mm^3^. Mice were randomized into six groups: Lipo-DPPC, Lipo-DOPE, 1h-RBC-DPPC, 1h-RBC-DOPE, 4h-RBC-DPPC and 4h-RBC-DOPE. These groups received an equivalent 5 mg/kg PTX dose three times a week, a total of four times of administration.

### Small animal *in vivo* imaging system optical imaging

Fluorescence was measured *ex vivo* using an *in vivo* imaging system (IVIS) Spectrum (PerkinElmer, Caliper IVIS Lumina LT S3). DiI-stained liposomes were prepared according to the protocol of DiI dye kit (Beyotime, China), which were added to RBCs, and then incubated at 37°C at different time. The samples were injected into C57 mice through the tail vein (PTX amount fixed at 5 mg/kg), and then mice were sacrificed at different time points. The corresponding organs and blood samples were dissected and imaged by IVIS. An excitation wavelength of 549 nm and an emission wavelength of 565 nm were chosen in unmixing mode. The total radiant efficiency (p/s/cm^2^/sr/μW/cm^2^) was determined using Living Image software (PerkinElmer).

### Statistical analysis

All statistical analyses were performed using SPSS 7.0. All data were expressed as the mean ± standard deviation. For normally distributed data, the significance of mean differences was determined using unpaired Student’s *t*-test between two groups or ANOVA followed by Newman–Keuls multiple comparison test among multiple groups. A difference was considered statistically significant if the *P*-value was <0.05.

## Results and discussion

### RBCs and liposomes combined DDS

**Figure 1.**
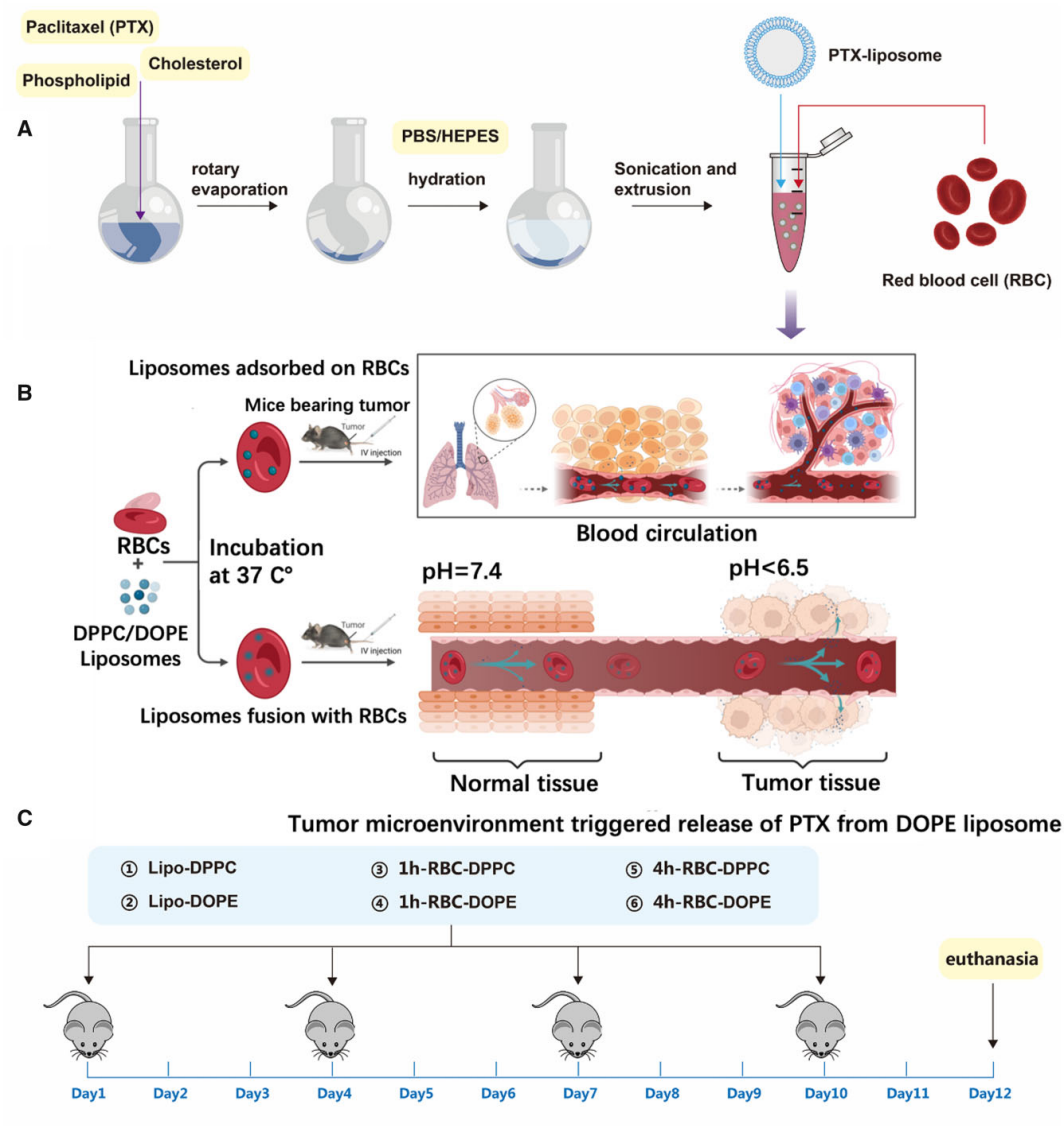
Schematic design of RBCs and liposomes combined DDS for anti-tumor therapy. (**A**) Liposomes were prepared by the conventional thin-film method. (**B**) Schematic diagram of liposomes combined with RBCs—by adsorption or fusion. First, liposomes are mixed with washed RBCs, which causes the liposomes to bind to the surface of the RBCs. The RBC–liposome complexes are then injected into a blood tail vessel. For liposomes adsorbed onto RBC, the RBC–liposome complex does not bump into the walls of large blood vessels, but when the RBC enters the capillaries, whose lumens are smaller than the diameter of RBCs, the liposomes adsorbed onto RBCs are pressed against the capillary wall, leading to transfer of the liposomes to the endothelial cells of the capillary. Some of them are taken up by the endothelial cells, while the other are flushed out by the blood flow and then enter the blood circulation again [20]. For pH-sensitive RBC–DOPE liposomes, these complexes respond to the low pH tumor microenvironment by destabilizing the lipid layer of the liposome and releasing the drug cargo in the tumor site. The liposomes fused with RBC do not detach from RBC during passing through the capillary, but instead, this RBC–liposome hybrid DDS continuously releases the drug (PTX diffusion out from the lipid bilayers) in the blood for the tumor therapy. (**C**) An experimental LLC subcutaneous transplantation tumor model was employed to study the anti-tumor effect. Treatment started when tumors were 90–110 mm^3^. Mice were randomized into six groups: Lipo-DPPC, Lipo-DOPE, 1h-RBC-DPPC, 1h-RBC-DOPE, 4h-RBC-DPPC and 4h-RBC-DOPE. These groups received an equivalent 5 mg/kg PTX dose three times a week, a total of four times of administration (PTX: 5 mg/kg; administered every 3 days, *n* = 8).

Conventional liposomes injected intravenously are rapidly cleared from the circulation by the RES. It is proved that opsonization of liposomes and their complement- or Fc-receptor-mediated uptake by macrophages in the liver and spleen accounts for this clearance [31, 32]. RBC represents a nearly ideal carrier for drug delivery within the vascular system as they have a long blood circulation half-life [33]. In the basic research of blood, human RBCs normally have a lifespan of 100–120 days (of note, mouse RBC lifetime is about a third of human’s counterpart). The volumes of human RBCs and mouse RBCs were ∼90 and ∼50 μm^3^, respectively. Previous studies have proved that major differences in RBCs characteristics between rabbit, mouse and human could significantly impact the PLGA NPs attachment efficacy and biocompatibility. In order to improve the potential for clinical translation of RBC DDS, we have chosen the human RBCs and designed the human RBC/liposome composite DDS, in which RBCs would carry the PTX-liposomes to circulate in blood. This novel DDS would enhance liposome’s circulation half-life and tumor accumulation amount. PTX-encapsulated liposomes were first fabricated using the lipid thin-film method. The combination ways (adsorption or fusion) between liposomes and RBCs were modulated by the incubation time [34, 35]. After the incubation of PTX-liposomes (PTX-lipo) with RBCs *in vitro*, the RBC–liposomes (RBC-lipo) were injected via the vein tail of tumor-bearing mice to study the tumor therapeutic effect, blood circulation time of RBC-lipo and organ accumulation *in vivo* (Fig. 1).

### Characterization of liposomes and RBCs–liposomes

**Figure 2.**
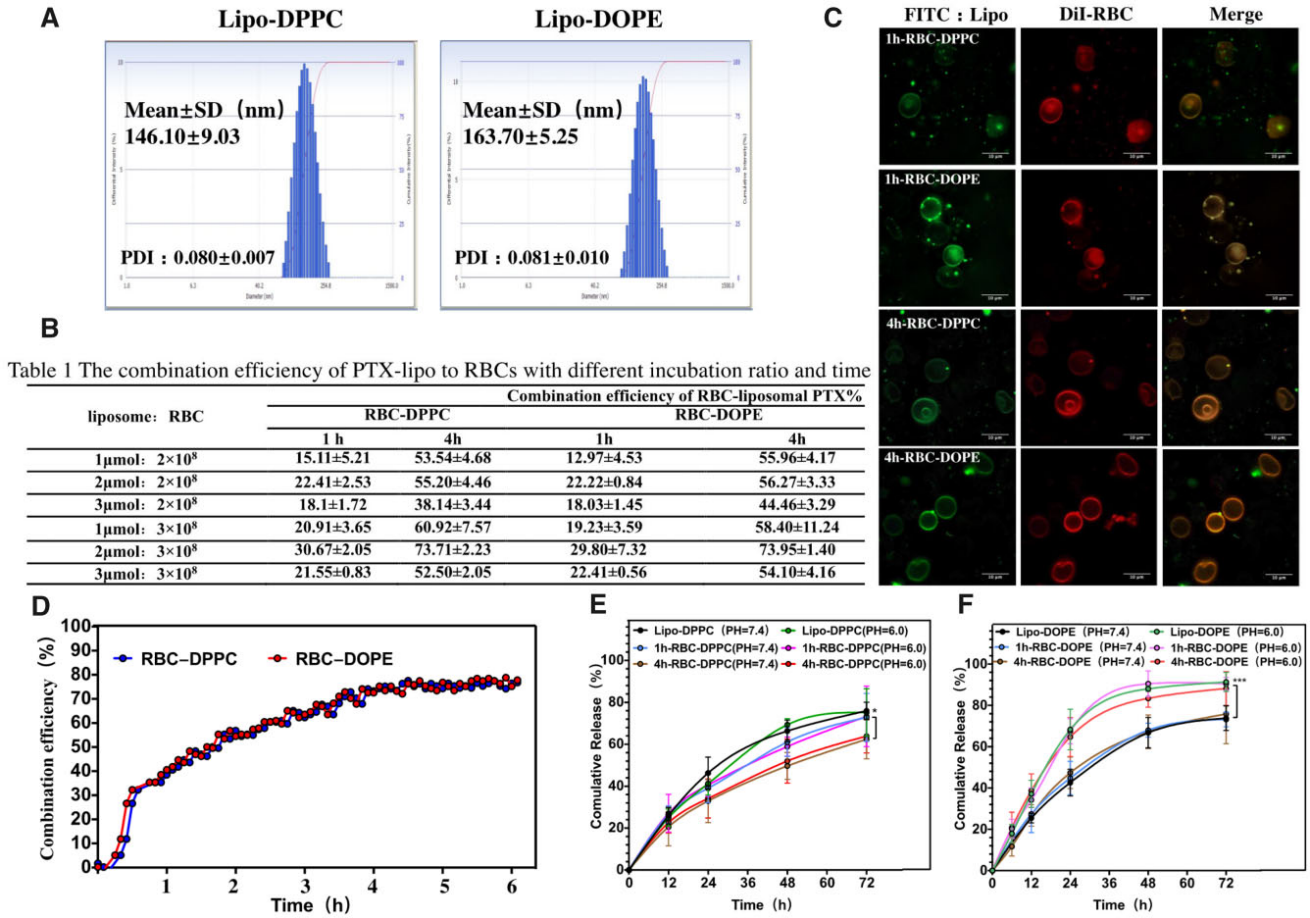
Characterization of liposomes (Lipo-DPPC/Lipo-DOPE) and RBC-lipo. (**A**) DLS size of liposomes (Lipo-DPPC/Lipo-DOPE). (**B**) The combination efficiency of liposomes to RBCs with different incubation ratio and time (*n* = 5). (**C**) Confocal images; green: FITC-liposomes; red: DiI-RBCs; scale bar: 10 μm. (**D**) Kinetics of combination between RBC and liposomes by FRET. Fluorescence intensity was measured at excitation and emission wavelengths of 464 and 522 nm, respectively. (**E** and **F**) Drug release of PTX from liposomes and RBC-lipo in PBS (pH = 6.0/7.4) (*n* = 5) (*P < 0.5; **P < 0.01; **P < 0.001).

Previous studies on liposome–cell interactions have shown that different formulations of liposomes can interact with different cell types to various degrees [36]. In this study, we prepared two kinds of PTX-encapsulated liposomes and studied their combination efficiencies with RBCs. The PTX encapsulation efficiencies in DPPC and DOPE liposomes are 91.14 ± 1.84% and 89.14 ± 0.70%, respectively. These two kinds of liposomes (Lipo-DPPC and Lipo-DOPE) were characterized in terms of hydrodynamic size and poly-dispersity index (PDI). Figure 2A shows that the sizes of the Lipo-DPPC and Lipo-DOPE are 146.1 ± 9.03 and 163.7 ± 5.25 nm, while both types of liposomes have narrow size distributions (PDI < 0.1). The combination mode between (adsorption/fusion) RBCs and liposomes can be modulated by the incubation time.

At 37°C, RBC membranes are in a liquid–crystalline state. With the increased fluidity of phospholipids, it creates favorable conditions for liposome–cell aggregation and fusion [37]. Thus, 37°C was selected as the interaction temperature between RBCs and liposomes. Figure 2B and Table 1 show the combination efficiency between these liposomes and RBCs, which indicated that the ratio of 2 μmol of liposomes with 3 × 10^8^ RBCs could obtain the highest drug loading amount. The combination efficiencies of Lipo-DPPC and Lipo-DOPE incubated with RBCs for 1 h were 30.67 ± 2.05% and 29.80 ± 7.32%, respectively; the combination efficiencies of Lipo-DPPC and Lipo-DOPE incubated with RBCs at 4 h were 73.71 ± 2.23% and 73.95 ± 1.40%, respectively.

**Table 1.** The combination efficiency of PTX-lipo to RBCs with different incubation ratio and time

	Combination efficiency of RBC-liposomal PTX%			
Liposome:RBC	RBC-DPPC		RBC-DOPE	
	1 h	4 h	1 h	4 h
1 μmol:2 × 10^8^	15.11 ± 5.21	53.54 ± 4.68	12.97 ± 4.53	55.96 ± 4.17
2 μmol:2 × 10^8^	22.41 ± 2.53	55.20 ± 4.46	22.22 ± 0.84	56.27 ± 3.33
3 μmol:2 × 10^8^	18.1 ± 1.72	38.14 ± 3.44	18.03 ± 1.45	44.46 ± 3.29
1 μmol:3 × 10^8^	20.91 ± 3.65	60.92 ± 7.57	19.23 ± 3.59	58.40 ± 11.24
2 μmol:3 × 10^8^	30.67 ± 2.05	73.71 ± 2.23	29.80 ± 7.32	73.95 ± 1.40
3 μmol:3 × 10^8^	21.55 ± 0.83	52.50 ± 2.05	22.41 ± 0.56	54.10 ± 4.16

To study the co-localization of liposomes with RBCs, FITC-lipid incorporated liposomes were prepared and RBCs were stained with lipophilic DiI dye. After incubation of liposomes with RBCs for 1 or 4 h, RBCs were washed gently with PBS and then imaged with confocal microscope. Figure 2C revealed that both liposomes and RBCs were observed co-localized suggesting a successful combination between liposomes and RBCs.

Unlike other cells, mature RBCs are generally unable to endocytose NPs (including liposomes). RBCs are routinely subjected to large deformations as the cell circulates throughout the body and squeezes through capillaries. To maintain the structure during the deformation and high flow rate, the large flexibility of the RBCs is primarily attributable to the cell membrane. Erythrocyte membrane is composed of a lipid bilayer and a protein skeleton containing mostly of actin and spectrin. Those special protein skeleton membrane decrease membrane mobility in mature RBCs and limits endocytosis [38]. In most cases, the combination between liposomes and mature RBCs should be either attachment or fusion. Fusion means the integration of lipid bilayers of the liposomes to the RBCs’ membrane. To study the combination between liposomes and RBCs, FRET experiments have been done and shown in Fig. 2D. When these liposomes were incubated with RBCs for 1 h, the combination efficiencies of RBC-DPPC and RBC-DOPE increased to 38.5% and 40.57%, respectively. Therefore, it is highly likely that the combination mode between RBCs and liposomes is more likely to be nonspecific adsorption onto RBC surface during the short period of incubation time. And some adsorbed liposomes on RBC surface may have lipid and cholesterol transfer with RBC membrane. Stoll *et al.* [15] have reported similar results when they incubate liposomes with RBCs. When the RBCs and liposomes were incubated together for 4 h, the combination efficiencies of RBC-DPPC and RBC-DOPE were 75.28% and 76.47%, respectively. These results suggest that more liposomes may fuse with the RBCs membrane.

In Fig. 2E and F, the *in vitro* release profile showed that the two types of liposomes loaded RBCs (RBC-DPPC/DOPE) exhibited similar release of PTX in PBS with pH 7.4. While the Lipo-DOPE and 1h/4h-RBC-DOPE showed rapid release characteristics both in PBS with pH 6.0, the results verified that DOPE liposome was pH-sensitive and could release the encapsulated drug faster under acidic conditions. Furthermore, DOPE is an unsaturated lipid, while DPPC is a saturated lipid [39], the DOPE liposomes would release PTX faster that the DPPC liposomes [40]. However, there were differences between the release profiles of 1h-RBC-DPPC and 4h-RBC-DPPC. In a previous study [41], lipophilic fluorophore R18-labeled liposomes were incubated with RBCs to investigate the interaction mechanism. Their results proved that the transfer of R18 from liposome to RBCs was due to both liposome adsorption and fusion. The merger of both membranes and lateral diffusion of liposomal lipids into the RBC lipid membrane bilayers resulted in spatial separation of membrane R18 fluorophores and therefore, a dequenching effect, marked by a significant increase of the RBC mean fluorescence intensity. Thus, we speculated that some PTX in DPPC may diffuse into the cytosol of RBCs during the fusion, which would delay the release of PTX.

### Characterization of RBCs after combination with liposomes

**Figure 3.**
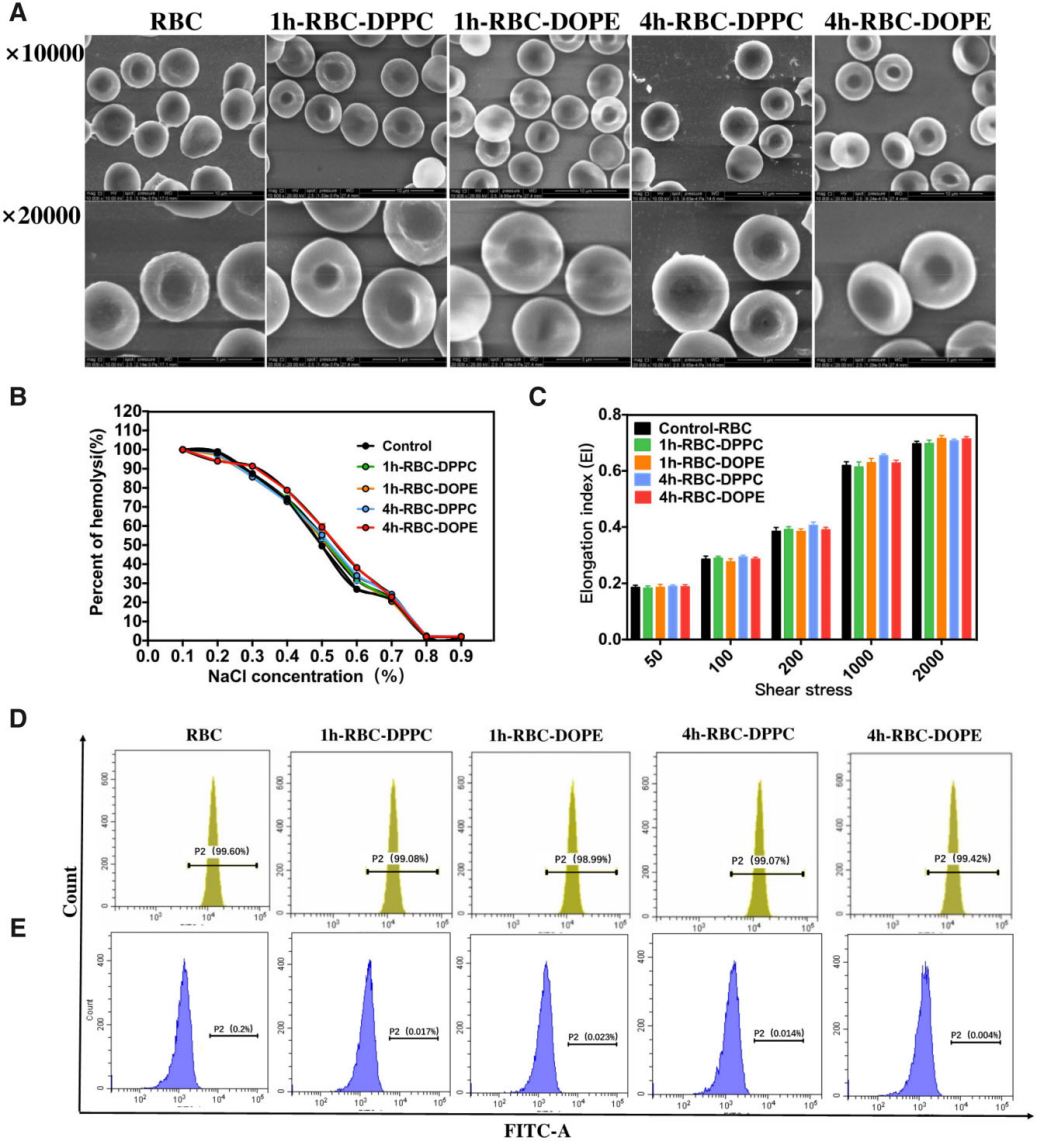
The effects of liposomes on RBCs. (**A**) SEM images of RBC combined with liposomes, scale bar: 5–10 μm. (**B**) Osmotic fragility of 1h-RBC-DPPC, 4h-RBC-DPPC, 1h-RBC-DPPC, 4h-RBC-DPPC and the RBC control (*n* = 5). (**C**) RBC deformability of 1h-RBC-DPPC, 4h-RBC-DPPC, 1h-RBC-DPPC, 4h-RBC-DPPC and the RBC control (*n* = 5). (**D**) CD47 expression of the control RBCs and different types of 1h/4h-RBC-lipo. (**E**) The exposure of PS on the control RBCs and 1h/4h-RBC-lipo. The results of positive control for the expression of CD47 and exposure of PS are shown in Supplementary Fig. S1.

As is known to all, the biconcave shape and corresponding deformability of the RBCs is an essential feature of its biological function [42]. This feature of RBCs may be affected during the incubation with liposomes. Membrane change of RBCs can cause quick removal from the circulation predominantly by the spleen and liver, which may affect the efficacy of RBC-based DDS [43, 44]. Therefore, it is imperative to investigate whether it is any morphological/physiological changes of RBCs occurred after the adsorption or fusion with liposomes. Based on the current study, there was no significant difference between RBCs control and liposome-treated RBCs in terms of RBC hemolysis rate (Table 2). Furthermore, the morphology of RBCs and 1h/4h-RBC-lipo was studied using SEM and it was shown in Fig. 3A. The SEM images proved that liposomes combined RBCs maintained their native concave structure like the control RBCs. The osmotic fragility and deformability of RBCs were also studied to evaluate any changes in the RBCs membrane after incubating procedure. The RBCs control released 50% of hemoglobin at the sodium chloride concentration of 0.510 ± 0.002%, whereas RBCs–liposomes released the same amount of hemoglobin at the same salt concentration (Fig. 3B). Likewise, there was no significant difference between control RBCs and liposome-treated RBCs for RBC deformability (Fig. 3C).

**Table 2.** Comparison of hemolysis rates after incubation of various liposomes with erythrocyte (*n* = 5)

	Control-RBC	1h-RBC-DPPC	1h-RBC-DOPE	4h-RBC-DPPC	4h-RBC-DOPE
Hemolysis (%)	0.021 ± 0.003	0.021 ± 0.002	0.021 ± 0.004	0.021 ± 0.006	0.020 ± 0.004

RBC membrane damage is evaluated by quantification of the amount of PS and CD47 on the RBC membrane for the loss of CD47 and an increase of PS eversion can induce the fast clearance of RBCs [45]. As is known to all, RBCs stored at 4°C for 1 month would show an increase in hemolysis, the loss of CD47, and an increase in PS exposure [46]. Thus, RBCs preserved for 30 days were employed as the positive control group for RBC damage. According to Supplementary Fig. S1 and Supplementary Table S1, damaged RBCs showed that the surface membrane protein CD47 decreased to 54.09%, the positive rate of PS reached 8.38% and the hemolysis rate increased to 0.73%. In Fig. 3D and E, there were no differences between control (fresh) RBCs and liposome-treated RBCs for the CD47 and PS amount, indicating that the incubation of liposomes with RBCs has a limited effect on RBCs. Taken together, these two types of liposomes used in this study do not affect the original characteristics and morphology of RBCs after the combination with liposomes, indicating that this liposome incubation approach has little effect on RBCs.

### Organ enrichment and blood circulation time of liposomes and 1-h/4-h-RBC-lipo *in vivo*

**Figure 4.**
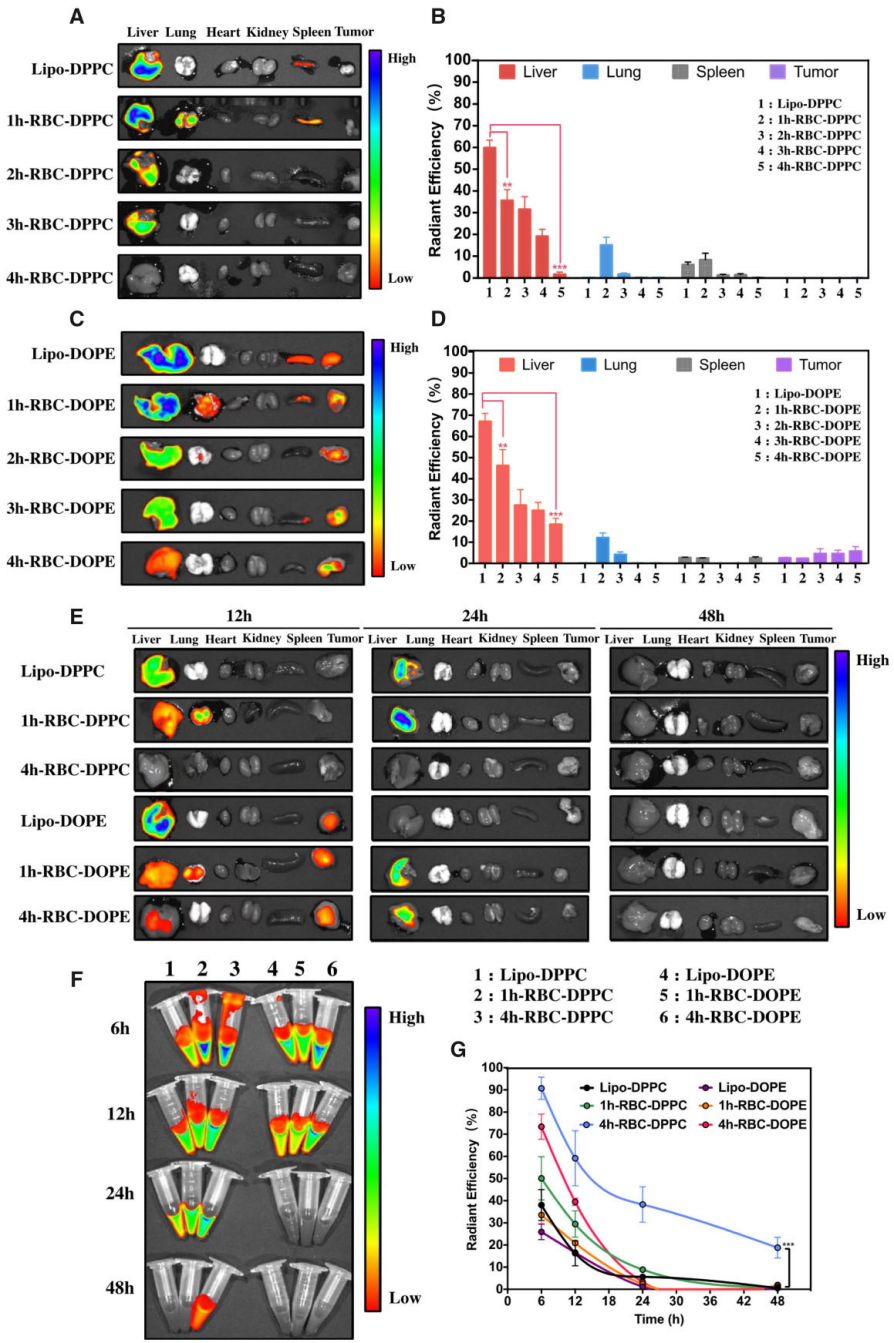
(**A**) The biodistribution of DiI-labeled DPPC liposomes and DPPC liposomes combined RBC with different incubation time, while the mice were sacrificed after IV injection for 6 h. (**B**) Quantification of accumulation of DiI-labeled Lipo-DPPC or RBC-DPPC (incubation time is 1–4 h) for 6 h by IVIS after intravenously injected for 6 h (*n* = 3) (*P < 0.05). (**C**) The biodistribution of DOPE and RBC-DOPE with different incubation time. (**D**) Quantification of organs accumulation of DiI-labeled Lipo-DOPE or RBC-DOPE (incubation time is 1–4 h) for 6 h by IVIS (data are presented as means ± SD) (*n* = 3) (*P < 0.05). (**E**) Representative *ex vivo* imaging of biodistribution of the C57BL/6J mice intravenously injected with DiI-labeled liposomes or 1h/4h-RBC-Lipo, while the mice were sacrificed at different time points. (**F**) Images of the blood samples of C57BL/6J at different time points by IVIS. (**G**) Quantification of DiI fluorescently intensity in the blood over time (*n* = 5; mean ± SD) (**P < 0.01; **P < 0.001).

The DiI-labeled liposomes incubated with RBCs were injected through the tail vein of the mice to investigate their biodistribution *in vivo* using IVIS. Figure 4A and C revealed that the control groups (Lipo-DPPC/Lipo-DOPE) were rapidly accumulated in the liver and spleen *in vivo* after injection for 6 h. Several previous studies [23, 47] have reported similar results and proved that most of the liposomes were cleared by the phagocytes in RES system within a couple of hours upon injection, which was one of the major limitations of liposomal DDS. To address this issue, liposomes were combined with RBCs at different time followed by IV injection to mice in order to reduce the clearance in RES, prolonging their blood circulation and enhancing the PTX therapeutic effect. In this study, two types of liposomes DPPC and DOPE liposomes were prepared, respectively. To enhance the PTX accumulation in the tumor, the pH-responsive DOPE lipid was incorporated into DPPC liposome, in which the liposomes would dissociate in the acidic tumor microenvironment, thus releasing the encapsulated drugs. As shown in Fig. 4, both 1h-RBC-DPPC and 1h-RBC-DOPE showed that 15.16 ± 3.56% and 12.20 ± 2.16% of the liposomes could accumulate in the lung. The reason for such accumulation in the lung was that liposomes were shedding off from the RBCs surface while RBCs (diameter 7–9 μm) repeatedly squeezed through the capillaries (1–5 μm) in the lung, i.e. the RH effect [48, 49]. Previous studies [19] have well demonstrated that nanocarriers (PLGA NPs, nanogel or PS NPs) adsorbed onto RBCs (with the incubation duration of 30 min to 1 h) possessed the characteristics of RH behavior. While the incubation time between liposomes and RBCs was increased from 2 to 4 h, the liposome may start to fuse with RBCs and showed no enrichment in the lung [50]. Although RH provides a promising targeting strategy for lung disease, there is still liver and spleen deposition for the RH liposomes (1h-RBC-DPPC/DOPE) as shown in Fig. 4A, C and E. The possible reason was that detached RH liposomes from the RBCs, which were not effectively transferred to endothelial cells and marginated leukocytes in the lung capillaries, may circulate in the bloodstream alone followed by clearance by the RES system. Thus, improvement of internalization of NPs in the lung endothelium is crucial for RH-based DDS.

Figure 4C and D showed both Lipo-DOPE and 1h/4h-RBC-DOPE accumulation at the tumor site. With the increment of incubation time, there were lesser DOPE liposomes accumulated in the liver and spleen. When the incubation time increases from 1 to 4 h, the fluorescence ratio at the liver of 4h-RBC-DOPE group decreases from 67.16 ± 3.72% to 23.51 ± 2.86%. Interestingly, our results showed that the 4h-RBC-DPPC was not enriched in any organs. To systematically study the liposome enrichment and blood circulation life *in vivo*, the mice were injected with either DiI-stained liposomes or RBC-lipo and sacrificed at 12, 24 and 48 h, respectively. Figure 4E–G showed that both DPPC and DOPE liposomes enriched less in the liver and spleen with increasing the incubation time, while the fluorescence ratio of liposomes in the blood revealed the opposite trend. It could be due to the fact that the fusion of liposomes with RBCs membrane was more stable and thus the liposomes were not detached from RBCs in the lung capillaries, resulting in extending the half-life of liposomes in the blood circulation as well as reducing the clearance by RES.

DPPC, a saturated lipid, is more stable than the unsaturated DOPE lipid [51]. DOPE are stable at physiological pH, but it undergoes destabilization induced by acidic pH in the tumor, thus leading to the release of the encapsulated drugs in the tumor microenvironment [52]. In Fig. 4E, Lipo-DOPE, 1h-RBC-DOPE and 4h-RBC-DOPE were found to possess 5.78 ± 1.18%, 6.44 ± 1.74% and 8.76 ± 2.01%, respectively, tumor accumulation after IV for 6 h. Although PEGylated liposomal system has a half-life for 36–48 h, they still suffered from lowered cellular uptake, poor tumor targeting and accelerated blood clearance upon repeated administration. Therefore, we did not use PEG-lipids for liposome fabrication. In addition, DPPC liposomes or RBC-DPPCs do not exhibit tumor accumulation but possess longer blood circulation. Interestingly, we found that 4h-RBC-DPPC showed prolonged blood circulation time as shown by IVIS data (Fig. 4F and G). After 48 h, there was still fluorescence signal for 4h-RBC-DPPC (fluorescence ratio: 19.81 ± 4.66%) in blood, indicating that the fusion of Lipo-DPPC with RBCs membrane could effectively avoid the clearance of RES system and thus obtain the prolonged blood circulation *in vivo*. The fluorescence ratio of the DOPE groups was lower than 1% after circulating for 24 h, indicating that the DOPE groups were cleared faster *in vivo* than the DPPC groups (Fig. 4E–G). In summary, the low pH in the tumor would trigger the drug release of DOPE liposomes, which caused the different behavior of DPPC and DOPE liposomes *in vivo*.

### Therapy efficacy of tumor-bearing mice

**Figure 5.**
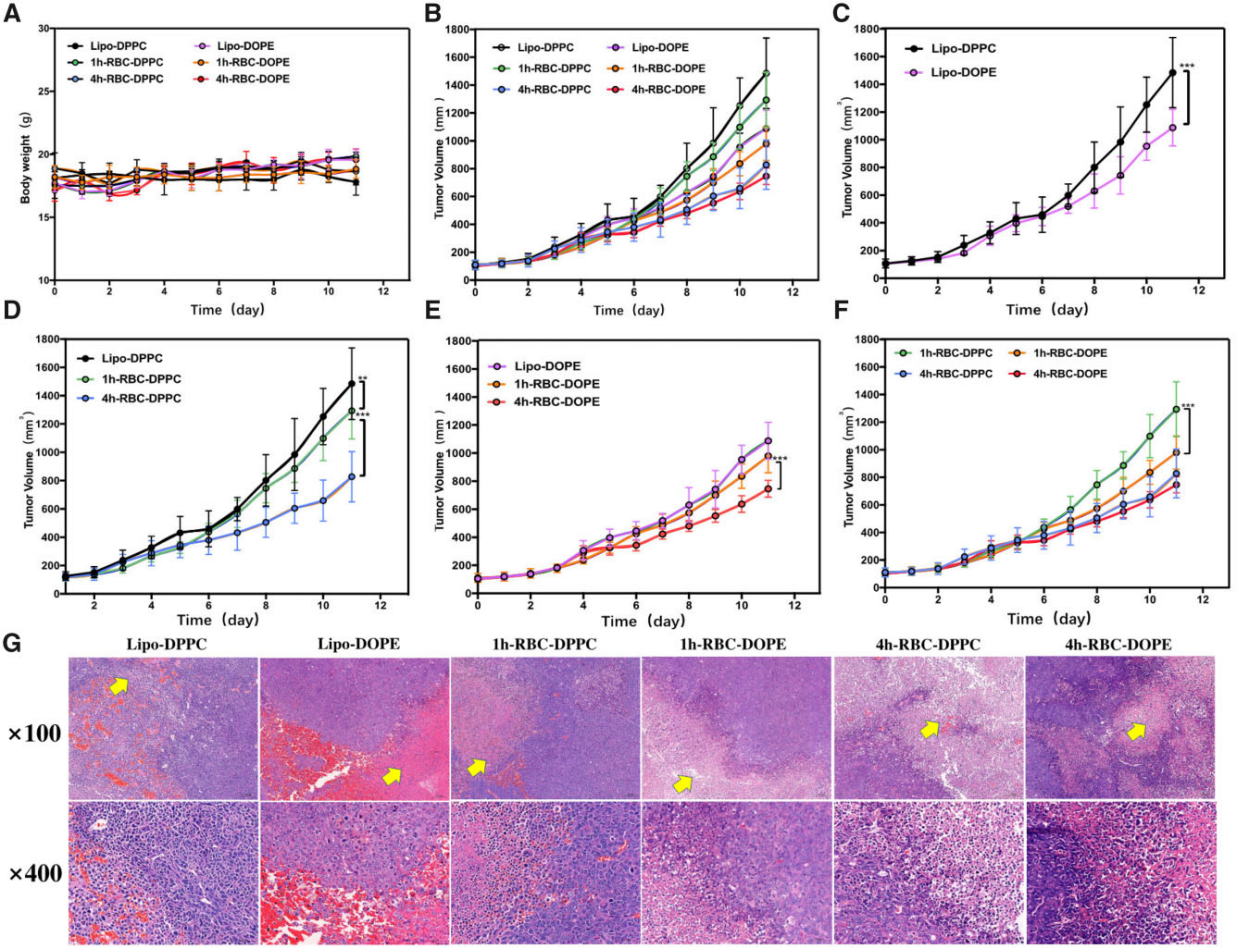
(**A**) C57BL/6J Mice body weight monitoring overtime (*n* = 8). (**B**) Tumor progression was closely monitored by weekly tumor volume measurement using a caliper (PTX 5 mg/kg, *n* = 8). (**C**) Tumor growth curves in mice injected with liposome (Lipo-DPPC/DOPE) alone every 3 days via the tail vein (*n* = 8). (**D**) Tumor growth curves in mice injected with Lipo-DPPC and RBC-DPPC every 3 days via the tail vein (*n* = 8). (**E**) Tumor growth curves in mice injected with Lipo-DOPE and RBC-DOPE every 3 days via the tail vein (*n* = 8). (**F**) Tumor growth curves in mice injected with RBC-DPPC and RBC-DOPE every 3 days via the tail vein (*n* = 8). (G) Histopathologic analyses of HE-stained tissue sections from tumor site after the indicated treatment. Yellow triangular arrows represent the necrosis (*P < 0.5; **P < 0.01; **P < 0.001).

The inhibition of tumor growth was studied in tumor-bearing mice and the results are shown in Fig. 5. There was no significant difference in the body weight of mice in each group, indicating that the RBC/liposome combined DDS was safe for mice (Fig. 5A). Tumor pictures are shown in Supplementary Fig. S2. Figure 5B–F showed that the tumor growth rate of RBC-lipo groups was significantly lower than that of the liposome-only groups. Among them, the tumor growth rate of the Lipo-DOPE group was significantly slower than that of the Lipo-DPPC groups, due to tumor targeting ability in the acidic tumor microenvironment (Fig. 5C). As shown in Fig. 5D, 4h-RBC-DPPC presented the best anti-tumor growth effect among the three groups. The reason may be explained by longer blood circulation half-life result shown in Fig. 4. These results indicated that 4h-RBC-DPPC could effectively avoid the clearance by RES system, and thus enhance the therapeutic effect. Figure 5E showed similar results as Fig. 5D, in which RBC-lipo systems are more effective in reducing tumor progression. In addition, the better therapeutic effect of 4h-RBC-lipo than 1h-RBC-lipo was probably due to their longer half-life in the blood; and interestingly there was no difference in the tumor growth rate between the 4h-RBC-DPPC group and the 4h-RBC-DOPE group (Fig. 5F). Although the DOPE group had obvious tumor targeting at the initial hours, the fluorescence ratio of DOPE groups *in vivo* (Fig. 4F) disappeared after circulating for 24 h, indicating that they were unable to sustain long circulation in the blood. While the 4h-RBC-DPPC group could circulate in the blood for more than 48 h, continuingly releasing the drug cargo during the systemic blood circulation, though there was no tumor enrichment being observed. Our results suggested that the anti-tumor therapy efficacy was a synergistic effect of both long blood circulation life and tumor accumulation ability. Based on the current study, fusion DPPC-RBC DDS may be a promising therapeutic way for hematologic tumors as it would keep circulating in the blood and without organ accumulation. Whereas the fusion DOPE-RBC DDS may be a good candidate for the therapy of solid tumor, as it exhibits a significantly higher amount of drug accumulation in the tumor (about 5–8%) compared with that of the conventional liposomes DDS (0.7% of the drug in the tumor). In summary, the RH technique or RBC fusion approach for liposome-based DDS exhibited an improved therapeutic effect compared with the traditional liposomal DDS.

Figure 5G shows the pathological section images of the different treatment groups. The tumor necrosis area of lipo-DPPC/DOPE both was 30%, while that of 1h-RBC-DPPC/DOPE was 40% and 50%, respectively. 4h-RBC-DPPC/DOPE achieved 70% versus 80% tumor necrosis area, respectively, and was accompanied by apoptosis. Both 4h-RBC-DPPC/DOPE groups were able to inhibit tumor growth substantially and achieved enhanced anti-tumor growth curve. Taken together, the results showed that the tumor inhibitory effect of liposomes could be significantly improved by modulating the combination of liposomes and RBCs. The efficacy of RBC-lipo prepared by the fusion approach was significantly better than that of the adsorption group.

## Conclusion

The combining of drug-loaded liposomes with RBCs can effectively enhance the blood circulation life, reduce the RES clearance and improve liposome’s anti-tumor therapeutic effect. Our study proved that the anti-tumor therapy efficacy was a synergistic effect of both long blood circulation life and tumor accumulation ability. Thus, fusion DPPC-RBC DDS, which shows relatively low tumor accumulation, but has a long blood circulation time, may be a promising therapeutic way for hematologic tumors, while fusion DOPE-RBC DDS is a good candidate for the therapy of solid tumor, as it exhibits about 5–8% tumor accumulation. Liposomes adsorbed on RBC showed lung accumulation property, which may be a new way for lung cancer therapy. In summary, RBC–liposome combined DDS is promising for the anti-cancer treatment using autologous RBCs.

## Supplementary Material

rbad045_Supplementary_Data
