# Supplementary material for: Hybrid liposome–erythrocyte drug delivery system for tumor therapy with enhanced targeting and blood circulation
Source: Regen Biomater. 2023 Apr 26;10:rbad045. doi: 10.1093/rb/rbad045 (PMC10224802; doi:10.1093/rb/rbad045)
Supplement: rbad045_Supplementary_Data [file rbad045_supplementary_data.docx]

**Supporting Information**

Table S1 Comparison of hemolysis rate between normal and damaged RBC

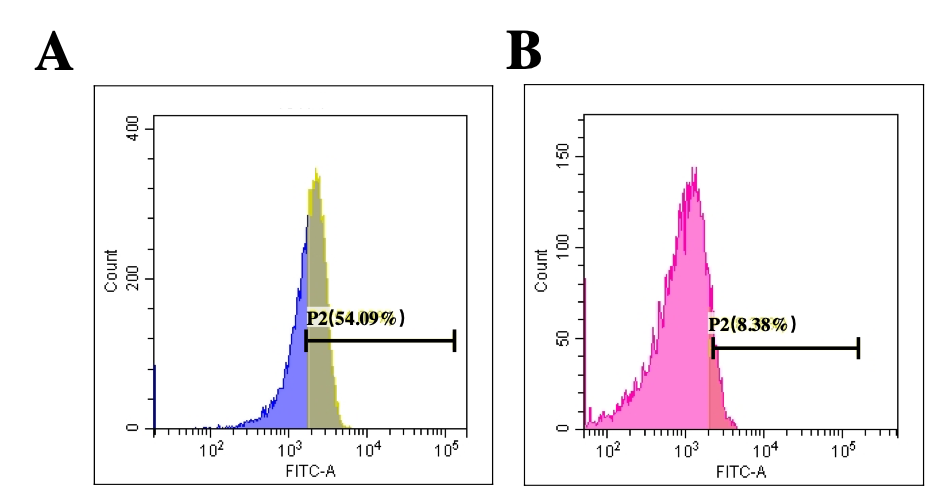


Figure S1 Positive control of RBC characteristics (A) CD47 and (B) PS expression of the damaged RBCs.

Figure S2 Tumor graphs of each group.
